# The Institutional Worker

**Published:** 1912-02-03

**Authors:** 


THE
### INSTITUTIONAL WORKER
**Being a Special Supplement to "The Hospital"**

### Editor's Notices.

**Contributions:**

Contributions should be written, or preferably typed, on one side of the paper only, and all articles sent in are accepted upon the distinct understanding that they are forwarded to THE HOSPITAL only.

The Editor cannot undertake to return MSS. not used, but when a stamped directed envelope is enclosed the MSS. may be returned if a special request is made.

Accepted articles and paragraphs of news will be paid for after publication at the scale rate.

**Address:**

To prevent delay all contributions and letters on editorial business must be addressed exclusively to the Editor, "The Hospital," 29 Southampton Street, Strand, London, W.C. It is important that this regulation shall be strictly observed.

**Correspondence:**

Correspondence on all subjects is invited. The name and address of correspondents must be given as a guarantee of good faith, but not necessarily for publication.

**Special Articles:**

Special articles are invited, and questions, inquiries, and paragraphs upon all matters relating to the work, administration, and management of general, special, mental, fever, cottage and convalescent hospitals, sanatoria, homes, institutions, societies and organisations for the treatment or care of the sick, injured and dependents of all classes. Every matter affecting the interests and welfare of the staff of all grades working in these institutions will receive special consideration.

**Administrative Medicine, Research and Items of News:**

Special terms will be paid for approved articles on subjects in this wide field by experts who have made some section of it their special study and interest. We wish to give prominence to every movement tending to advance accuracy of diagnosis, efficiency in treatment, and the development of modern methods to eradicate disease and promote the welfare of the suffering.

**A Bureau of Information:**

We invite inquiries and applications for information and help in providing for that numerous class of sufferers who require special care or house-room which they cannot obtain in their own homes or secure for themselves. We cannot, however, prescribe or recommend practitioners.

**Books for Review:**

Publishers are particularly requested to send advance proofs of any new books of importance whenever possible, as the Editor has made arrangements to publish immediate reviews on a new plan.

**Photographs, Plans, Blocks, and Illustrations:**

It is requested that wherever possible MSS. may be accompanied by illustrations in any of the above forms. The name of the sender and of the article to which it belongs should be written on the back of each photograph, drawing, or block for purposes of identification.

**Local Papers:**

Newspapers containing reports or news paragraphs should be marked and addressed to the Sub-Editor.

**The Coupon System:**

A coupon will be found at the bottom of the third inside page of the cover each week. This coupon must be attached to every question or inquiry to which an answer is desired in THE HOSPITAL.

### Manager's Notices.

Letters relating to the publication, sale, and advertising departments of THE HOSPITAL must be addressed to the Manager, "The Hospital," The Hospital Building, 28 and 29 Southampton Street, Strand, London, W.C.

**Subscriptions** which may begin at any time, are payable in advance. Cheques and Post-Office orders, crossed London County and Westminster Bank, Covent Garden branch, should be made payable to the Manager of THE HOSPITAL and addressed to him at The Hospital Building, 28 and 29 Southampton Street, Strand, London, W.C.

**Advertisements:**

All advertisements for THE HOSPITAL must reach the Manager not later than Wednesday morning in each week. For scale of charges see pages v and 2.

**Institution Authorities.**

THE economical management of an Institution depends very greatly upon purchasing supplies in the cheapest market.

IT is often impossible locally to secure the highest quality at the lowest prices.

IN consequence, a section will be reserved in this part of the paper for announcements of Institutions inviting Tenders for Contracts.

THIS will enable Authorities of Institutions to secure tenders for supplies from all over the country, and ensure purchases at the lowest possible prices consistent with quality.

THE charge for Tenders is 6s. per inch, and for this small outlay many pounds may be saved on supplies of all descriptions.

**Special Rates** will be quoted for those institutions which agree to send all their vacancy, contract, and public notices to THE HOSPITAL each year, so as to make it a file of such announcements for ready reference.

**Classified Advertisements:**

All small advertisements will be classified, for this section of the paper is widely read and of special interest. We wish to encourage those wanting appointments who are attracted to institutional work, and therefore offer them the reduced rate of *twenty-one words and under for* 1s., and *for every ten and part of ten words beyond*, 6*d*.

This form may be used for small prepaid advertisements, and when filled up should be posted to the

*Manager*, THE HOSPITAL,
28 and 29 Southampton Street,
Strand, W.C.

Appointments Wanted, 21 words ... 1s. 0d.
Appointments Vacant, 21 words ... 2s. 6d.

### OFFICIAL APPOINTMENTS VACANT.

### Poor-Law Vacancies.

Assistant Engineer (£35), Edmonton Union. Mr. F. Shelton, C. to G., White Hart Lane, Tottenham. Feb. 3.
Assistant Female Attendant (£25), Bath Union. Mr. W. E. Winchworth, C. to G., Bath. Feb. 7.
Assistant Male Attendant (£25), Bath Union. Mr. W. E. Winchworth, C. to G., Bath. Feb. 7.
Assistant Nurses (£25), Parish of Liverpool Convalescent Home for Boys. G. W. Coster, Vestry Clerk, Parish Offices, Liverpool. Feb. 9.
Assistant Nursery Attendant (£17), Holborn Union. Matron of the Workhouse, 1a Shepherdess Walk, N.
Assistant Nursery Attendant (£21 18s.), St. George-in-the-East Guardians. Mr. R. M. Lochner, C. to G., Raine Street, Old Gravel Lane, E. Feb. 6.
Assistant Overseer (Valuer) (£500), Birmingham T.C. The City Treasurer, Birmingham. Feb. 10.
Bathman and Barber (£28), Hunslet Union. Mr. F. W. Mee, C. to G., Hunslet. Feb. 7.
Boys' Male Attendant and Yardsman (£28 18s.), St. George-in-the-East Guardians. Mr. R. M. Lochner, C. to G., Raine Street, Old Gravel Lane, E. Feb. 6.
Case-Paper Clerk (£100), Lewisham Union. Mr. H. C. Mott, C. to G., 394 High Street, Lewisham, S.E. Feb. 5.
Case-Paper Clerk (£80), St. Giles and Bloomsbury Guardians. Mr. J. Appleton, C. to G., 57 Broad Street, Bloomsbury, W.C. Feb. 5.
Case-Paper Clerk (£100), Birkenhead Union. Mr. J. Carter, C. to G., Birkenhead. Feb. 6.
Charge Nurse (£30), Rochford Union. Mr. F. Gregson, C. to G., Southend-on-Sea. Feb. 5.
Charge Nurse (£32), Northampton Union. Mr. W. Fawkes, C. to G., Northampton. Feb. 9.
Charge Nurse (£32), Rochdale Union. Mr. R. A. Leach, C. to G., Rochdale. Feb. 17.
Charge Nurse (£30), Norwich Guardians. Mr. E. J. W. Huggins, C. to G., Norwich.
Charge Nurse (£30), Oldham Union. H. A. Quarmby, C. to G., Rochdale Road, Oldham. February 13.
Charge Nurses (two) (£32), Mutford and Lothingland Union. E. S. Norton, C. to G., 148 London Road, Lowestoft. Feb. 9.
Children's Attendant (£30), Coventry Union. Mr. E. H. Evans, C. to G., Coventry. Feb. 9.
Female Assistant Relieving Officer (£100), Paddington Guardians. Mr. H. F. Aveling, C. to G., 313-319 Harrow Road, W. Feb. 12.
Female Assistant Relieving Officer and Infant Protection Visitor (£50), Sevenoaks Union. F. H. Vibert, C. to G., Bank Chambers, Sevenoaks. Feb. 14.
Female Attendant (£24), Cardiff Union. Mr. A. J. Harris, C. to G., Cardiff. Feb. 12.
Foster-Mother (£25), Nuneaton Union. Mr. C. Blakeway, C. to G., Nuneaton. Feb. 5.
Foster-Mother (£28), Huddersfield Union. Mr. E. A. Rigby, C. to G., Huddersfield.
Foster-Mothers (3) and Relief Foster-Mother (£27 each), Tynemouth Union. Mr. Tom Percival, C. to G., North Shields. Feb. 7.
Foster-Parents (married couple) (£30 and £20), Chelmsford Union. Mr. A. S. Duffield, C. to G., Chelmsford. Feb. 8.
Head Girls' Attendant (£28 18s.), St. George-in-the-East Guardians. Mr. R. M. Lochner, C. to G., Raine Street, Old Gravel Lane, E. Feb. 6.
Head Laundress (£1 weekly), Edmonton Union. Mr. F. Shelton, C. to G., White Hart Lane, Tottenham. Feb. 3.

HEAD NURSERY ATTENDANT (£28 18s.), St. George-in-the-East Guardians. Mr. R. M. Lochner, C. to G., Raine Street, Old Gravel Lane, E. Feb. 6.
HOUSE MOTHERS (£24), Kensington and Chelsea School District. Mr. H. D. Aslett, Clerk to the Managers, King Street, Hammersmith, W. Feb. 8.
HOUSE PORTER (£30), Southampton Union. Mr. A. J. Walden, C. to G., Southampton. Feb. 3.
INFANTS' NURSE (£27 10s.), Nottingham Guardians. Mr. G. M. Howard, C. to G., Nottingham. Feb. 8.
JUNIOR ASSISTANT CLERK (15s. weekly), Lewisham Union. Mr. H. C. Mott, C. to G., 394 High Street, Lewisham, S.E. Feb. 5.
LABOUR MASTER (£30), Paddington Guardians. Mr. H. F. Aveling, C. to G., 313-319 Harrow Road, W. Feb. 7.
MALE ATTENDANT (£30), Aston Union. Mr. J. North, C. to G., Vauxhall Road, Birmingham. Feb. 5.
MALE ATTENDANT (£28), Macclesfield Union. Mr. F. May, C. to G., Macclesfield. Feb. 5.
MALE NURSE (£35), Bermondsey Guardians. Mr. E. Pitts Fenton, C. to G., 283 Tooley Street, S.E. Feb. 8.
MALE SICK ATTENDANT (£35), Edmonton Union. Mr. F. Shelton, C. to G., White Hart Lane, Tottenham. Feb. 3.
MATERNITY SISTER (£35), Oldham Union. H. A. Quarmby, C. to G., Rochdale Road, Oldham. February 13.
MATRON'S ASSISTANT (£20), Bakewell Union. Mr. A. Hawes, C. to G., Bakewell. Feb. 10.
NIGHT CHARGE NURSE (£30), Whitechapel Union. Mr. F. J. Tootell, C. to G., 74 Vallance Road, N.E. Feb. 7.
NIGHT SISTER (£35), Stockport Union. Matron of the Workhouse, Shaw Heath, Stockport.
NURSE (£25), Blean Union. Mr. J. E. Burch, C. to G., 39 Castle Street, Canterbury. Feb. 5.
NURSE (£25), Lutterworth Union. Mr. T. C. Bodycote, C. to G., Lutterworth. Feb. 7.
NURSE (£27 10s.), Stockbridge Union. Matron of the Workhouse, Stockbridge.
NURSE (£30), Pewsey Union. Mr. R. Dixon, C. to G., Pewsey. Feb. 6.
NURSE (£30), Isle of Thanet Union. C. Taylor, C. to G., Minster, nr. Ramsgate. Feb. 22.
NURSES (4) (£30), Gateshead Union. Mr. G. Craighill, C. to G., Gateshead. Feb. 3.
NURSERY ATTENDANT (£23), Wokingham Union. Mr. J. F. Sargeant, C. to G., Wokingham. Feb. 5.
PORTER AND PORTRESS (married couple) (£35 joint), East Retford Union. Mr. J. G. Dimock, C. to G., East Retford.
PORTER, ETC., AND ASSISTANT TO MATRON (married couple) (£30 and £25), Kendal Union. Mr. A. Milne, C. to G., Kendal.
PORTRESS, ETC. (£25), Chelsea Guardians. Mr. C. W. Shepherd, C. to G., 250 King's Road, Chelsea, S.W. Feb. 3.
PROBATIONERS (£10), Wakefield Union. H. Beaumont, C. to G., 47 Kirkgate, Wakefield. Feb. 13.
PROBATIONER NURSES (£10), Parish of Brighton. B. Burfield, C. to G., Parochial Offices, Brighton.
PROBATIONER NURSES (£10), Steyning Union. A. Flowers, C. to G., Union Offices, Shoreham-by-Sea, Sussex.
PROBATIONER NURSES (£13), West Ham Union. T. Smith, C. to G., Union Road, Leytonstone, N.E.
RELIEVING OFFICER, VACCINATION OFFICER, ETC. (£90 and fees, about £50), Newton Abbot Union. Mr. F. Horner, C. to G., Newton Abbot. Feb. 6.
SCULLERYMAID (£19 18s.), St. George-in-the-East Guardians. Mr. R. M. Lochner, C. to G., Raine Street, Old Gravel Lane, E. Feb. 6.
SECOND LAUNDRYMAID (£18), Edmonton Union. Mr. F. Shelton, C. to G., White Hart Lane, Tottenham. Feb. 3.
SCULLERYMAID (£18), Edmonton Union. Mr. F. Shelton, C. to G., White Hart Lane, Tottenham. Feb. 3.
SISTER (£33), Sheffield Union. Matron, Union Hospital, Fir Vale, Sheffield. Feb. 3.
SISTER-IN-CHARGE (£35), Nottingham Guardians. Mr. G. M. Howard, C. to G., Nottingham. Feb. 8.
STAFF NURSE (£31), West Ham Union. T. Smith, C. to G., Union Road, Leytonstone. Feb. 14.
WARD SISTER (£36), West Ham Union. T. Smith, C. to G., Union Road, Leytonstone. Feb. 14.
WARDMAID (£14), Edmonton Union. Mr. F. Shelton, C. to G., White Hart Lane, Tottenham. Feb. 3.

### Institutional and Administrative Vacancies.

MATRON, Aberdeen Royal Infirmary. A. Scott Finnie, Clerk and Treasurer, 343 Union Street, Aberdeen. Feb. 3.
MATRON (£50), Pontypridd and District Cottage Hospital. G. B. Williams, Old Bank Chambers, Pontypridd.
NIGHT SISTER (£35), Preston Royal Infirmary. Matron.
NIGHT SUPERINTENDENT AND HOME SISTER (£45), Bristol General Hospital. Matron. Feb. 5.
OUT-PATIENT SISTER AND MASSEUSE (£35), Victoria Hospital for Sick Children, Hull. Matron. Feb. 10.
SISTER (£30), Chesterfield and North Derbyshire Hospital. Matron.
STAFF NURSE (£24), Bangor (Co. Down) Hospital. The Hon. Secretaries. Feb. 3.
STAFF NURSE (£20), Evelina Hospital for Children, Southwark. Matron.

### Municipal Vacancies.

ACCOUNTANT (£200), Bexley U.D.C. Mr. T. G. Baynes, Clerk to the Council, Bexley Heath. Feb. 7.
ASSISTANT NURSE (£26), Isolation Hospital, Tivington, Minehead. T. H. Hosegood, Clerk to District Hospital Committee, 18 The Avenue, Minehead. Feb. 10.
ASSISTANT NURSE (£20), Leek and D. Council Isolation Hospital. Clerk to the Council, Town Hall, Leek.
ASSISTANT NURSE (£26), Newhaven Urban District Council Isolation Hospital. E. Knightley, Clerk to Council, Newhaven. Feb. 6.
ASSISTANT SURVEYOR (£130), Easington R.D.C. Mr. J. M. Longden, Clerk to the Council, Easington. Feb. 14.
CLERK OF WORKS (Underground Conveniences) (£3 3s. weekly), Tottenham U.D.C. Mr. W. H. Prescott, Engineer, Council Offices, Tottenham. Feb. 5.
CORRESPONDENCE CLERK (£70), Middlesex C.C. Mr. W. G. Austin, Clerk to the Council, 63 Victoria Street, S.W. Feb. 5.
ENGINEERING ASSISTANT (£3 3s. weekly), Darlington T.C. The Borough Surveyor, Darlington.
FEMALE HEALTH VISITORS (two) (£80), Hemsworth Rural District Council. J. Scholefield, Clerk, Hemsworth, near Wakefield. February 13.
HEALTH VISITOR (£100), Bethnal Green B.C. Mr. C. G. E. Fletcher, Town Clerk, Bethnal Green. Feb. 12.
HIGHWAY SURVEYOR (£110), Glanford Brigg R.D.C. Mr. F. C. Hett, Clerk to the Council, Brigg.
INSPECTOR OF NUISANCES (£130), Gwyrfai R.D.C. Mr. J. H. Thomas, Clerk to the Council, 14 Market Street, Carnarvon. Feb. 8.
INSPECTOR OF NUISANCES, ETC. (£105), Darlaston U.D.C. Mr. J. Corbett, Clerk to the Council, Darlaston. Feb. 12.
INSPECTORS OF MEAT, ETC. (2) (£3 weekly), Birmingham T.C. Mr. E. V. Hiley, Town Clerk, Birmingham. Feb. 12.
MATRON (£50), Birmingham T.C. Hospital. The Medical Officer of Health, Birmingham. Feb. 10.
NURSE (£30), Weston-super-Mare U.D.C. Infectious Diseases Hospital. S. C. Smith, Clerk to Council, Town Hall, Weston-super-Mare. Feb. 6.
NURSES (£30), Kingston-upon-Hull Infectious Diseases Hospital. Chairman, Hospitals Committee, Town Hall, Hull. Feb. 13.
NURSE-MATRON (£41 12s.), Seaham Harbour U.D. Council Infectious Diseases Hospital. H. B. Wright, Clerk, Council Offices, Seaham Harbour. Feb. 9.
NURSE-MATRON AND PORTER, ETC. (married couple) (£65 joint), Saffron Walden Joint Hospital Board. Mr. W. Adams, Clerk to the Board, 14 Church Street, Saffron Walden. Feb. 20.

